# Epidermal growth factor receptor gene copy number aberration at the primary tumour is significantly associated with extracapsular spread in oral cancer

**DOI:** 10.1038/bjc.2011.22

**Published:** 2011-02-08

**Authors:** C Michikawa, N Uzawa, H Sato, Y Ohyama, N Okada, T Amagasa

**Affiliations:** 1 Maxillofacial Surgery, Maxillofacial Reconstruction and Function, Division of Maxillofacial and Neck Reconstruction, Graduate School, Tokyo Medical and Dental University, 1-5-45 Yushima, Bunkyo-ku, Tokyo 113-8549, Japan; 2 Diagnostic Oral Pathology, Oral Restitution, Division of Oral Health Sciences, Graduate School, Tokyo Medical and Dental University, Tokyo, Japan

**Keywords:** extracapsular spread, oral cavity, squamous cell carcinoma, fluorescence *in situ* hybridisation, lymph node

## Abstract

**Background:**

Extracapsular spread (ECS) of lymph node metastasis in head and neck cancers, including oral squamous cell carcinomas (OSCCs), is known to reflect tumour aggressiveness, and is significantly associated with high rates of loco-regional recurrence, distant metastasis, and poor outcome. The purpose of this study was to confirm ECS as an important prognostic indicator and to determine the significant factors associated with ECS in OSCCs.

**Methods:**

We investigated the incidence of ECS and impact of ECS on survival in 127 OSCC patients. To determine the factors significantly correlated with ECS, we examined many factors, including the clinicopathological features of primary tumours, lymph node metastasis, and copy number aberrations of the cyclin D1 gene (*CCND1*) and epidermal growth factor receptor gene (*EGFR*) at primary tumours, and evaluated the value of predicting the risk of ECS of the metastatic lymph node.

**Results:**

Kaplan–Meier and multivariate disease-free and overall survival analysis clearly demonstrated that ECS is an independent prognostic factor in OSCCs. Moreover, logistic regression analysis showed that the number of pathologically positive nodes and copy number aberrations of *EGFR* at the primary tumour are independent predictors of ECS.

**Conclusions:**

The findings suggest that ECS is an independent prognostic factor in OSCCs. Moreover, the number of pathologically positive lymph nodes and *EGFR* numerical aberrations of the primary tumour were also shown to be excellent predictors of ECS in OSCCs. Preoperative evaluation of *EGFR* numerical aberrations might therefore be a useful tool for selecting patients at high risk of ECS, who would benefit from targeted aggressive multimodality therapy.

Head and neck squamous cell carcinoma (HNSCC), including oral cancer, is the sixth most common malignancy in humans. Despite tremendous improvements in surgery, radiotherapy, and chemotherapy over the past decade, the prognosis for patients with HNSCCs has more or less remained unchanged for the past 30 years (Forastiere *et al*, 2001; Silverman, 2001). This is primarily because patients continue to die from metastatic disease at regional and distant sites, although local recurrence and second primary tumours are also a cause of death in these patients. It is widely accepted that the presence of metastasis in cervical lymph nodes is a reliable prognostic indicator of survival and disease recurrence (Kalnins *et al*, 1977; Schuller *et al*, 1980; Snow *et al*, 1982; Grandi *et al*, 1985). In addition to nodal metastasis, extracapsular spread (ECS) of lymph node metastasis is significantly associated with a further increase in loco-regional recurrence and distant metastasis, further reducing the overall survival (OS) rate of patients with this disease (Noguchi *et al*, 1999; Prim *et al*, 1999; Myers *et al*, 2001; Greenberg *et al*, 2003; Woolgar *et al*, 2003; Wenzel *et al*, 2004; Larsen *et al*, 2009). The presence of ECS is therefore a marker for biologically aggressive disease, and is the most pivotal predictor of survival, recurrence, and distant metastasis. Thus, patients who present with ECS require intensification of multimodality therapy. However, the detection of ECS is currently performed by histological examination of the dissected lymph nodes after surgery, making it impracticable to determine ECS of lymph node metastasis preoperatively. Therefore, to determine patients with ECS, who would benefit from invasive treatment, it is necessary to determine the predictive markers that indicate a high risk of ECS.

Although ECS is frequently present in patients with advanced nodal metastasis, it is also detected not only in a small number of patients with early-nodal disease and but also even in those with a clinically negative (N0) neck (Alvi and Johnson, 1996; Coatesworth and MacLennan, 2002). This has made prediction of ECS even more difficult. Improvements in various imaging modalities, such as computed tomography, magnetic resonance imaging, ultrasound, and positron emission tomography have allowed us to detect lymph node metastasis and ECS preoperatively. However, all these imaging methods are limited in their ability to detect smaller tumour volume (Sano and Myers, 2007). The incidence of micrometastases that cannot be detected by any imaging technique is as high as 25%, and thus, on the basis of imaging criteria alone, it is still difficult to detect ECS occurring in early-nodal disease or N0 neck (Connor and Olliff, 2000).

New diagnostic strategies for the prediction of ECS are therefore urgently required to identify patients with aggressive disease. Identification of predictive biomarkers that indicate a high risk of ECS would have a major role in determining the optimal therapeutic strategy for these aggressive diseases and in achieving a high survival rate. Unfortunately, however, there are currently no molecular markers for the detection of ECS in oral squamous cell carcinomas (OSCCs).

The cyclin D1 gene (*CCND1*) is a proto-oncogene, located on chromosome 11q13, that is elevated in response to extracellular mitogens. It is also a rate-limiting regulator of G1-phase progression through the cell cycle, and has been shown to be associated with an increase in lymph node stage in anterior tongue cancer (Motokura *et al*, 1991; Sherr, 1995; Bova *et al*, 1999; Carlos de Vicente *et al*, 2002). Since 1998, we have been investigating alterations in *CCND1* using fluorescence *in situ* hybridisation (FISH), with fine-needle aspiration (FNA) biopsy samples from primary OSCCs, and have demonstrated clearly that *CCND1* numerical aberrations are significantly associated with an invasive phenotype and cervical lymph node metastasis in OSCCs (Miyamoto *et al*, 2002, 2003; Myo *et al*, 2005; Uzawa *et al*, 2007; Takahashi *et al*, 2009). These observations suggest that *CCND1* might have an important role in the process of metastasis and the development of ECS.

On the other hand, the epidermal growth factor receptor gene (*EGFR*) is a transmembrane tyrosine kinase receptor. Signalling through this receptor leads to the activation of multiple signalling proteins that initiate a cascade of several signal pathways, including the Ras-Raf-mitogen-activated protein kinase pathway, the phosphatidylinositol 3-kinase-Akt pathway, and the signal transducer and activators of transcription pathway, all of which are potent oncogenic regulators of tumour cell growth, invasion, angiogenesis, and metastasis (Arteaga, 2002; Arany *et al*, 2003). Overexpression of *EGFR* is seen in approximately 80% of HNSCCs, and has been reported to be of strong prognostic value and to have a significant association with nodal metastasis (Rubin Grandis *et al*, 1998; Xia *et al*, 1999; Ang *et al*, 2002; Bei *et al*, 2004; Takahashi *et al*, 2009). However, EGFR overexpression is not always linked to the gene copy number aberrations (Chung *et al*, 2006; Egloff and Grandis, 2009). Thus, although our previous investigation revealed that copy number aberrations of this gene is one of the most significant prognostic markers in OSCCs (Takahashi *et al*, 2009), it is not clear that the association between EGFR gene copy number status and the presence of ECS. In the present study, we therefore focused on copy number changes in these two genes in primary tumours.

There are two aims of the present study. The first is to confirm the clinical and prognostic significance of the presence of ECS in OSCCs. The second is to determine the factors significantly correlated with the presence of ECS. We examine a number of factors including clinicopathological features of the primary tumour, lymph node metastasis, and the *CCND1* and *EGFR* gene status of primary tumours, and evaluate the value of predicting the risk of ECS of metastatic lymph nodes.

## Patients and methods

### Patient characteristics

The medical records of 127 consecutive OSCC patients who had undergone primary surgical excision with curative intent at the Maxillofacial Surgery, Graduate School, Tokyo Medical and Dental University (Tokyo, Japan), between June 1999 and April 2008, were reviewed for this study. No patients had preoperative treatment. All protocols of this study were reviewed and approved by the Research Ethics Committee of Tokyo Medical and Dental University. Informed consent was obtained from all patients in accordance with our Institutional guidelines. The clinical staging was defined on the basis of the International Union Against Cancer TNM classification (Sobin *et al*, 2009). The primary tumours were classified histopathologically as well-, moderately, or poorly differentiated according to the level of differentiation defined in the World Health Organization classification (World Health Organization, 1998). The mode of tumour invasion was also classified using a modification of the criteria of Jacobsson *et al* (Jacobsson *et al*, 1973; Yamamoto *et al*, 1983). Histological data obtained from neck dissection specimens included the number of positive nodes and the presence or absence of ECS. Extracapsular spread was defined as extension of the tumour through the capsule of the lymph node into the perinodal tissues. The presence of tumour cells in the capsule of the node was not considered ECS.

### Fine-needle aspiration–fluorescence *in situ* hybridisation analysis

To investigate the genetic status of the primary tumour, FISH analysis was performed. Samples were taken from 127 primary tumours by FNA technique, and FISH assays were performed as described previously using two types of BAC clone probes, specific for *CCND1* and *EGFR* (Vysis, Downers Grove, IL, USA), labelled with Spectrum Orange, and chromosome 11 and 7 centromeric DNA, labelled with Spectrum Green (Miyamoto *et al*, 2002). Samples were grouped as follows: balanced disomy, chromosome/nucleus ratio (C/N) ⩽2.5; balanced trisomy, C/N 26–3.0; balanced polysomy, C/N >3.0 (in which balanced patterns had an average ratio gene/chromosome copy number per nucleus (G/C) of 0.9–1.2); and amplification, G/C >1.2 and gene/nucleus ratio >3.0 (Sunpaweravong *et al*, 2005; Takahashi *et al*, 2009). Tumours showing disomy were considered normal, with all other tumours being considered to have gene numerical aberrations.

### Statistical analysis

Disease-free survival (DFS) and OS rates were estimated using the Kaplan–Meier method and statistical significance was determined using the log-rank test. Disease-free survival time was defined as the interval between the date of first visit and the date of the development of local, regional recurrence, and distant metastasis after surgery. Overall survival time was also calculated from the date of initial examination to the date of death, or to the date of the 5-year follow-up. Multivariate DFS and OS analyses were performed using the Cox proportional hazards model. Multivariate analysis for ECS risk factors was performed using the logistic regression analysis. The level of significance was set at *P*<0.05. All statistical analyses were performed using SPSS for Windows (version 17.0, SPSS, Inc., Chicago, IL, USA).

## Results

### Demographic data

A total of 127 patients (86 men (67. 7%); mean age 60.2 years; age range 20–89 years) were included in this study. The median follow-up period was 43.2 months (range 6.0–60 months).

### Tumour characteristics and staging

The sites of the primary tumours were derived from tongue (*n*=77), lower gingiva (*n*=27), floor of the mouth (*n*=10), upper gingiva (*n*=4), and the buccal mucosa (*n*=9). A total of 41 of the 127 patients (32.3%) had T1 tumours, 61 (48.0%) had T2 tumours, 12 (9.5%) had T3 tumours, and 13 (10.2%) had T4 tumours at the time of presentation. The growth pattern of the primary tumour was classified as superficial (*n*=27), exophytic (*n*=43), and endophytic (*n*=57). A total of 74 of the 127 patients (58.3%) had a N0 node, 21 (16.5%) patients had a pathologically positive node without ECS (pN+/ECS−), and 32 (25.2%) patients had a pathologically positive node with ECS (pN+/ECS+).

### Impact of extracapsular spread on survival

**Figure 1:**
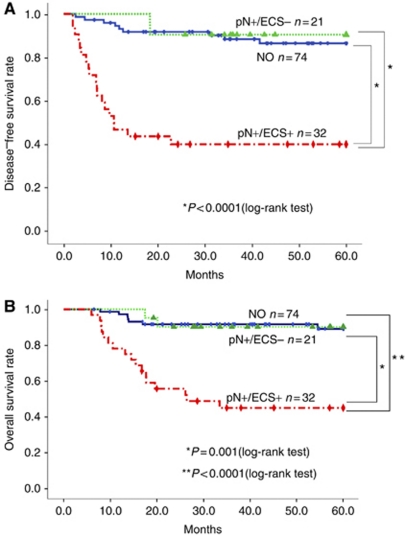
(**A**) Kaplan–Meier curve for disease-free survival according to lymph node status. (**B**) Kaplan–Meier curve for overall survival according to lymph node status.

To assess the impact of ECS on survival, the survival data of the patients were compared between the three groups, N0, pN+/ECS−, and pN+/ECS+. Kaplan–Meier survival curves for DFS and OS are presented in Figures 1A and B, respectively. Disease-free survival rates for 5 years are 86.5% for N0, 90.5% for pN+/ECS−, and 40.1% for pN+/ECS+ patients. Overall survival rates for 5 years are 89.1% for N0, 90.2% for pN+/ECS−, and 45.1% for pN+/ECS+ patients. These results clearly demonstrate the adverse impact of group pN+/ECS+ compared with groups N0 and pN+/ECS− on both disease recurrence (log-rank test. *P*<0.0001 and *P*<0.0001) and OS (log-rank test. *P*<0.0001 and *P*=0.001). Multivariate Cox proportional hazards analysis revealed that smoking habit, pathological T stage, cellular differentiation, and presence of ECS were independently significant in predicting DFS (Table 1). With regard to OS, the pathological T stage and presence of ECS retained a statistically significant prognostic value (Table 1). Therefore, pathological T stage and presence of ECS are significant independent predictors in both DFS and OS.

### Associations with extracapsular spread

The correlations between presence of ECS and clinicopathological parameters in the OSCC patients are summarised in Table 2. There were no significant associations between presence of ECS and age, smoking habit, tumour site, growth pattern, pathological T stage, and cellular differentiation. In contrast, the presence of ECS was significantly correlated with the number of pathologically positive nodes (*P*=0.001), indicating that ECS is more frequent in patients with multiple positive nodes. With regard to the size of the metastatic lymph node, although the presence of ECS occurred more frequently in large nodes at both the minor and major axis compared with small metastatic lymph nodes, these differences were not statistically significant. On the other hand, the presence of *CCND1* and *EGFR* numerical aberrations of the primary tumour was significantly associated with the presence of ECS in metastatic lymph nodes (*P*=0.011 and 0.004, respectively). A multivariate logistic regression analysis, including clinicopathological factors, lymph node metastasis, and genetic status of the primary tumour, revealed that only the number of pathologically positive lymph nodes and the presence of *EGFR* numerical aberrations of the primary tumour were significantly independent predictors of ECS (odds ratio=9.400 and 8.206, 95% confidence interval=2.136–41.370, and 1.631–41.295, *P*=0.003 and *P*=0.011, respectively, hit ratio=73.1% Table 3).

## Discussion

For HNSCCs, including oral cancer, ECS has been shown to be a significant predictor of treatment outcome. Myers *et al* (2001) reviewed 266 patients with SCC of the tongue and determined a 5-year disease-specific survival rate of 88% for pN0 patients, 65% for patients with intranodal lymph node metastases, and 48% for patients with extranodal lymph node metastases. Wenzel *et al* (2004) also showed that OSCC patients with no positive nodes or positive nodes without ECS had nearly the same 5-year rates for being free from distant metastases (79, 82%), local recurrence (61, 67%), neck recurrence (84, 87%), and survival (67, 59%), whereas those with ECS had 1.5–2 times worse rates for every clinical parameter. Shingaki *et al* (1999) reported a 5-year disease-specific survival rate of 40% with and 72% without ECS for patients with oral cancer. In the current study, we also clearly demonstrated the adverse impact of group pN+/ECS+ compared with groups N0 and pN+/ECS− on both disease recurrence and OS. Moreover, multivariate Cox proportional hazard analysis revealed that pathological T stage and presence of ECS is significantly correlated with disease recurrence and survival. These findings are in keeping with the previous observations that ECS is a discriminatory and significant predictor for prognosis of patients with HNSCCs (Noguchi *et al*, 1999; Prim *et al*, 1999; Shingaki *et al*, 1999; Myers *et al*, 2001; Greenberg *et al*, 2003; Woolgar *et al*, 2003; Wenzel *et al*, 2004; Larsen *et al*, 2009).

Of first interest in this study was that multivariate analysis indicated the presence of ECS rather than the existence of a metastatic lymph node as a significant predictor of disease recurrence and poor prognosis. Rather than indicating the difference between N0 and N+, these findings show that differentiation between intra- and extra-capsular spread of the lymph node contains the essential discriminatory power. Recently, Shaw *et al* (2010) reviewed 400 OSCC patients, and reported a 5-year OS rate in ECS-positive patients of 23% compared with 52% in pN+/ECS− patients and with 65% in pN0 patients. They also found differences in survival curves to be highly significant between pN0, pN+/ECS−, and pN+/ECS+ (*P*<0.001, log-rank test). These findings are in accord with our results, indicating that the presence of ECS is a more significant predictor of recurrence and survival than the presence of cervical lymph node metastasis.

However, at present, the existence of ECS can only be clarified after neck surgery, thus preventing preoperative determination. Moreover, as a significant number of ECS are detected in early-nodal disease and even in patients with N0 neck, recent imaging modalities are unable to detect ECS before surgery (Alvi and Johnson, 1996; Connor and Olliff, 2000; Coatesworth and MacLennan, 2002; Sano and Myers, 2007). Therefore, it is necessary to determine the predictive markers that indicate a high risk of ECS. Thus, in this study, we attempted to determine significant factors related to ECS: multivariate logistic regression analysis revealed that number of pathologically positive lymph nodes and presence of *EGFR* numerical aberrations of the primary tumour were significantly independent predictors.

ECS is histopathologically documented in approximately 60% of patients with cervical metastases, and is generally thought to be related to nodal size (Coatesworth and MacLennan, 2002). However, although ECS has been detected in 60–100% of lymph nodes >3 cm in diameter, it has also been shown in 39–59% of nodes <3 cm and in 23% of nodes <1 cm (Puri *et al*, 2003). Moreover, ECS has been determined in patients with N0 neck, whereas the incident rate of ECS in those with N0 neck is between 13 and 60% (Cole and Hughes, 1997). In this study, we investigated the association between presence of ECS and metastatic lymph node size, and determined that although ECS occurs more frequently in larger-sized nodes, in both the minor and major axis, compared with small nodes; but these differences were not statistically significant. This finding is compatible with the previous observations (Cole and Hughes, 1997; Puri *et al*, 2003). Taken together, these findings suggest that ECS can occur in several ways. Puri *et al* (2003) recently speculated that in larger nodes, mechanical disruption, as a result of an expanding tumour mass, is the main cause of ECS. On the other hand, in smaller nodes, ECS might result from tumour emboli, lodged in the capsular sinuses, or by focal destruction of capsular collagen by collagenase produced by the tumour cells. However, these processes remain to be elucidated, and further investigation is warranted.

Several reports have documented the clinicopathological factors significantly correlated with ECS. Recently, Imre *et al* (2008) retrospectively studied 186 patients with laryngeal and hypopharyngeal cancer and, using multivariate analysis, documented that only number of (>3) lymph node metastases was a significant independent predictor of ECS. More recently, also by means of multivariate analysis, Shaw *et al* (2010) examined 400 patients with OSCCs and demonstrated that age >75 years, smoking, and heavy use of alcohol were independent predictors of ECS. Furthermore, Woolgar *et al* (2003), using univariate analysis, investigated 173 OSCC patients and found that many clinicopathological parameters, including gender, age, status of the resection margin, number of positive nodes, largest metastatic deposit, highest anatomical level of involvement, and pathological N stage, were significantly correlated with ECS. In the present study, we retrospectively examined 127 patients with OSCCs and multivariate analysis revealed that number of pathologically positive lymph nodes and presence of *EGFR* numerical aberrations at the primary tumours were significantly associated with ECS. The number of positive lymph nodes was shown to have a correlation in the abovementioned reports, suggesting it can be used in the prediction of ECS. However, the number of pathologically positive nodes is determined after surgery, and it is therefore impracticable to obtain this information preoperatively. Therefore, it is not useful for preoperative selection of patients at high risk of ECS.

On the contrary, second but most interesting and important finding of this study was that ECS in the metastatic lymph node is significantly associated with *EGFR* numerical aberrations at the primary tumours. This finding suggests that patients at high risk of ECS could be preoperatively determined by examining *EGFR* copy number changes in primary tumours by means of FISH. Although there are several reports (Chen *et al*, 2003; Zhou *et al*, 2006; Huang *et al*, 2009) that showed the protein or mRNA overexpression of EGFR significantly correlated with ECS in the OSCCs, this is the first study investigating the association between EGFR gene copy number at the primary tumour and the presence of ECS in the metastatic lymph node, and clearly demonstrating the significant correlation between them. Generally, the association between EGFR FISH status and protein or mRNA expression level still remains to be concluded (Chung *et al*, 2006; Egloff and Grandis, 2009). Thus, EGFR gene copy number aberrations have not always linked to overxepression of this mRNA or protein. Therefore, at present, the significant association between *EGFR* FISH status and ECS might not be related with *EGFR* alone. FISH-positive status may be a surrogate marker of generalised genetic instability in the tumour or of additional genes that are co-amplified with EGFR. However, how these mechanisms contribute to the occurrence of ECS remains unclear. Further investigations are required.

In conclusion, the present study clearly demonstrated that ESC is an independent prognostic factor of OSCCs. Moreover, number of pathologically positive lymph nodes and *EGFR* numerical aberrations of the primary tumours were also shown to be excellent predictors of ECS in OSCCs. Especially, copy number changes of the *EGFR* in the primary tumours were indicated as clinically useful for selection of patients at high risk of ECS who would benefit from targeted aggressive multimodality therapy.

## Figures and Tables

**Table 1:** Independent prognosis factors in multivariate Cox proportional hazards analysis

	**Disease-free survival**			**Overall survival**		
**Variables**	***P*-value**	**HR**	**95% CI**	***P*-value**	**HR**	**95% CI**
Age	NS	—	—	NS	—	—
Gender	NS	—	—	NS	—	—
Alcohol habit	NS	—	—	NS	—	—
Smoking habit	0.0070	3.365	1.387–8.165	NS	—	—
Pathological T stage	0.0001	5.505	2.417–12.540	0.0001	4.574	2.006–10.429
Cellular differentiation	0.0380	2.448	1.051–5.701	NS	—	—
Mode of invasion	NS	—	—	NS	—	—
pN	NS	—	—	NS	—	—
ECS	0.0001	7.345	3.269–16.501	0.0001	5.986	2.460–14.566
*CCND1* numerical aberration	NS	—	—	NS	—	—
*EGFR* numerical aberration	NS	—	—	NS	—	—

Abbreviations: CI=confidence interval; ECS=extracapsular spread; *EGFR*=epidermal growth factor receptor gene; HR=hazard ratio; NS=not significant; pN=pathologically positive node.

**Table 2:** Univariate analysis of potential predictors of ECS

**Clinicopathologic parameters**	*N*−*	pN+ ECS−**	pN+ ECS+***	***P*-value^a^**
*Gender*				
Male	45	14	27	0.023^b^
Female	29	7	5	
*Age (years)*				
⩽60	35	10	15	NS
>60	39	11	17	
*Alcohol habit*				
(−)	38	8	10	0.005^b^
(+)	33	13	21	
*Smoking habit*				
(−)	39	12	11	NS
(+)	32	9	20	
*Tumor site*				
Tongue	45	13	19	NS
Lower gingiva	18	4	5	
Floor of mouth	3	2	5	
Upper gingiva	2	1	1	
Buccal mucosa	6	1	2	
*Growth pattern*				
Superficial	18	3	6	NS
Exophytic	28	8	7	
Endophytic	28	10	19	
*Pathological T stage*				
1–2	65	14	23	0.042^c^
3–4	9	7	9	
*Cellular differentiation*				
Well/moderate	64	19	24	NS
Poor	10	2	8	
*Mode of invasion*				
1–3	53	13	14	0.009^b^
4C–D	21	8	18	
*Number of positive nodes* d				
1	—	13	5	0.001^e^
⩾2	—	8	27	
*Size of positive nodes*^f^ *(mm)*				
Minor axis (3–20)				
⩽10	—	13	16	NS
>10	—	8	16	
Major axis (5–27)				
⩽14	—	14	14	NS
>14	—	7	18	
*Gene status in primary tumor*				
*CCND1* numerical aberration				
(−)	56	16	12	0.0001^b^
(+)	18	5	20	0.011^e^
*EGFR* numerical aberration				
(−)	51	18	14	0.018^b^
(+)	23	3	18	0.004^e^

Abbreviations: ECS=extracapsular spread; *EGFR*=epidermal growth factor receptor gene; NS=not significant.

a The *P*-value was determined using the two-tailed Fisher exact test.

b ^*^ *vs* ^***^.

c ^*^ *vs* ^**^.

d Determined according to histopathological diagnosis.

e ^**^ *vs* ^***^.

f Detected by computed tomography.

**Table 3:** Independent predictors of ECS in multivariate logistic regression analysis

**Variables**	**OR**	**95% CI**	***P*-value**
Age	—	—	NS
Gender	—	—	NS
Alcohol habit	—	—	NS
Smoking habit	—	—	NS
Pathological T stage	—	—	NS
Cellular differentiation	—	—	NS
Mode of invasion	—	—	NS
Number of positive nodes	9.400	2.136–41.370	0.003
*CCND1* numerical aberration	—	—	NS
*EGFR* numerical aberration	8.206	1.631–41.295	0.011

Abbreviations: CI=confidence interval; ECS=extracapsular spread; NS=not significant; OR=odds ratio.

## References

[bib1] Alvi A, Johnson JT (1996) Extracapsular spread in the clinically negative neck (N0): implications and outcome. Otolaryngol Head Neck Surg 114: 65–70 8570253 10.1016/S0194-59989670285-1

[bib2] Ang KK, Berkey BA, Tu X, Zhang HZ, Katz R, Hammond EH, Fu KK, Milas L (2002) Impact of epidermal growth factor receptor expression on survival and pattern of relapse in patients with advanced head and neck carcinoma. Cancer Res 62: 7350–7356 12499279

[bib3] Arany I, Chen SH, Megyesi JK, Adler-Storthz K, Chen Z, Rajaraman S, Ember IA, Tyring SK, Brysk MM (2003) Differentiation-dependent expression of signal transducers and activators of transcription (STATs) might modify responses to growth factors in the cancers of the head and neck. Cancer Lett 199: 83–89 12963127 10.1016/s0304-3835(03)00345-8

[bib4] Arteaga CL (2002) Epidermal growth factor receptor dependence in human tumors: more than just expression? Oncologist 7(Suppl 4): 31–39 12202786 10.1634/theoncologist.7-suppl_4-31

[bib5] Bei R, Budillon A, Masuelli L, Cereda V, Vitolo D, Di Gennaro E, Ripavecchia V, Palumbo C, Ionna F, Losito S, Modesti A, Kraus MH, Muraro R (2004) Frequent overexpression of multiple ErbB receptors by head and neck squamous cell carcinoma contrasts with rare antibody immunity in patients. J Pathol 204: 317–325 15476268 10.1002/path.1642

[bib6] Bova RJ, Quinn DI, Nankervis JS, Cole IE, Sheridan BF, Jensen MJ, Morgan GJ, Hughes CJ, Sutherland RL (1999) Cyclin D1 and p16INK4A expression predict reduced survival in carcinoma of the anterior tongue. Clin Cancer Res 5: 2810–2819 10537346

[bib7] Carlos de Vicente J, Herrero-Zapatero A, Fresno MF, Lopez-Arranz JS (2002) Expression of cyclin D1 and Ki-67 in squamous cell carcinoma of the oral cavity: clinicopathological and prognostic significance. Oral Oncol 38: 301–308 11978554 10.1016/s1368-8375(01)00060-4

[bib8] Chen IH, Chang JT, Liao CT, Wang HM, Hsieh LL, Cheng AJ (2003) Prognostic significance of EGFR and Her-2 in oral cavity cancer in betel quid prevalent area cancer prognosis. Br J Cancer 89: 681–686 12915878 10.1038/sj.bjc.6601171 PMC2376917

[bib9] Chung CH, Ely K, McGavran L, Varella-Garcia M, Parker J, Parker N, Jarrett C, Carter J, Murphy BA, Netterville J, Burkey BB, Sinard R, Cmelak A, Levy S, Yarbrough WG, Slebos RJ, Hirsch FR (2006) Increased epidermal growth factor receptor gene copy number is associated with poor prognosis in head and neck squamous cell carcinomas. J Clin Oncol 24: 4170–4176 16943533 10.1200/JCO.2006.07.2587

[bib10] Coatesworth AP, MacLennan K (2002) Squamous cell carcinoma of the upper aerodigestive tract: the prevalence of microscopic extracapsular spread and soft tissue deposits in the clinically N0 neck. Head Neck 24: 258–261 11891957 10.1002/hed.10020

[bib11] Cole I, Hughes L (1997) The relationship of cervical lymph node metastases to primary sites of carcinoma of the upper aerodigestive tract: a pathological study. Aust NZ J Surg 67: 860–865 10.1111/j.1445-2197.1997.tb07613.x 9451342

[bib12] Connor SE, Olliff JF (2000) Imaging of malignant cervical lymphadenopathy. Dentomaxillofac Radiol 29: 133–143 10849539 10.1038/sj/dmfr/4600521

[bib13] Egloff AM, Grandis JR (2009) Improving response rates to EGFR-targeted therapies for head and neck squamous cell carcinoma: candidate predictive biomarkers and combination treatment with Src inhibitors. J Oncol 2009: 896407 19636423 10.1155/2009/896407 PMC2712676

[bib14] Forastiere A, Koch W, Trotti A, Sidransky D (2001) Head and neck cancer. N Engl J Med 345: 1890–1900 11756581 10.1056/NEJMra001375

[bib15] Grandi C, Alloisio M, Moglia D, Podrecca S, Sala L, Salvatori P, Molinari R (1985) Prognostic significance of lymphatic spread in head and neck carcinomas: therapeutic implications. Head Neck Surg 8: 67–73 4077553 10.1002/hed.2890080202

[bib16] Greenberg JS, Fowler R, Gomez J, Mo V, Roberts D, El Naggar AK, Myers JN (2003) Extent of extracapsular spread: a critical prognosticator in oral tongue cancer. Cancer 97: 1464–1470 12627511 10.1002/cncr.11202

[bib17] Huang SF, Chuang WY, Chen IH, Liao CT, Wang HM, Hsieh LL (2009) EGFR protein overexpression and mutation in areca quid-associated oral cavity squamous cell carcinoma in Taiwan. Head Neck 31: 1068–1077 19360742 10.1002/hed.21067

[bib18] Imre K, Pinar E, Oncel S, Calli C, Tatar B (2008) Predictors of extracapsular spread in lymph node metastasis. Eur Arch Otorhinolaryngol 265: 337–339 17899142 10.1007/s00405-007-0464-0

[bib19] Jacobsson PA, Eneroth GM, Killander D, Moeberger G, Martenssen B (1973) A histological classification and grading of malignancy in carcinoma of larynx. Acta Radiol 12: 1–18 10.3109/02841867309131085 4725642

[bib20] Kalnins IK, Leonard AG, Sako K, Razack MS, Shedd DP (1977) Correlation between prognosis and degree of lymph node involvement in carcinoma of the oral cavity. Am J Surg 134: 450–454 911028 10.1016/0002-9610(77)90376-2

[bib21] Larsen SR, Johansen J, Sorensen JA, Krogdahl A (2009) The prognostic significance of histological features in oral squamous cell carcinoma. J Oral Pathol Med 38: 657–662 19563504 10.1111/j.1600-0714.2009.00797.x

[bib22] Miyamoto R, Uzawa N, Nagaoka S, Hirata Y, Amagasa T (2003) Prognostic significance of cyclin D1 amplification and overexpression in oral squamous cell carcinomas. Oral Oncol 39: 610–618 12798405 10.1016/s1368-8375(03)00048-4

[bib23] Miyamoto R, Uzawa N, Nagaoka S, Nakakuki K, Hirata Y, Amagasa T (2002) Potential marker of oral squamous cell carcinoma aggressiveness detected by fluorescence *in situ* hybridization in fine-needle aspiration biopsies. Cancer 95: 2152–2159 12412169 10.1002/cncr.10929

[bib24] Motokura T, Bloom T, Kim HG, Juppner H, Ruderman JV, Kronenberg HM, Arnold A (1991) A novel cyclin encoded by a bcl1-linked candidate oncogene. Nature 350: 512–515 1826542 10.1038/350512a0

[bib25] Myers JN, Greenberg JS, Mo V, Roberts D (2001) Extracapsular spread. A significant predictor of treatment failure in patients with squamous cell carcinoma of the tongue. Cancer 92: 3030–3036 11753980 10.1002/1097-0142(20011215)92:12<3030::aid-cncr10148>3.0.co;2-p

[bib26] Myo K, Uzawa N, Miyamoto R, Sonoda I, Yuki Y, Amagasa T (2005) Cyclin D1 gene numerical aberration is a predictive marker for occult cervical lymph node metastasis in TNM stage I and II squamous cell carcinoma of the oral cavity. Cancer 104: 2709–2716 16265665 10.1002/cncr.21491

[bib27] Noguchi M, Kido Y, Kubota H, Kinjo H, Kohama G (1999) Prognostic factors and relative risk for survival in N1-3 oral squamous cell carcinoma: a multivariate analysis using Cox's hazard model. Br J Oral Maxillofac Surg 37: 433–437 10687900 10.1054/bjom.1999.0146

[bib28] Prim MP, De Diego JI, Hardisson D, Madero R, Nistal M, Gavilan J (1999) Extracapsular spread and desmoplastic pattern in neck lymph nodes: two prognostic factors of laryngeal cancer. Ann Otol Rhinol Laryngol 108(7 Part 1): 672–676 10435927 10.1177/000348949910800710

[bib29] Puri SK, Fan CY, Hanna E (2003) Significance of extracapsular lymph node metastases in patients with head and neck squamous cell carcinoma. Curr Opin Otolaryngol Head Neck Surg 11: 119–123 14515090 10.1097/00020840-200304000-00010

[bib30] Rubin Grandis J, Melhem MF, Gooding WE, Day R, Holst VA, Wagener MM, Drenning SD, Tweardy DJ (1998) Levels of TGF-alpha and EGFR protein in head and neck squamous cell carcinoma and patient survival. J Natl Cancer Inst 90: 824–832 9625170 10.1093/jnci/90.11.824

[bib31] Sano D, Myers JN (2007) Metastasis of squamous cell carcinoma of the oral tongue. Cancer Metastasis Rev 26: 645–662 17768600 10.1007/s10555-007-9082-y

[bib32] Schuller DE, McGuirt WF, McCabe BF, Young D (1980) The prognostic significance of metastatic cervical lymph nodes. Laryngoscope 90: 557–570 7359975 10.1288/00005537-198004000-00001

[bib33] Shaw RJ, Lowe D, Woolgar JA, Brown JS, Vaughan ED, Evans C, Lewis-Jones H, Hanlon R, Hall GL, Rogers SN (2010) Extracapsular spread in oral squamous cell carcinoma. Head Neck 32: 714–722 19827119 10.1002/hed.21244

[bib34] Sherr CJ (1995) D-type cyclins. Trends Biochem Sci 20: 187–190 7610482 10.1016/s0968-0004(00)89005-2

[bib35] Shingaki S, Nomura T, Takada M, Kobayashi T, Suzuki I, Nakajima T (1999) The impact of extranodal spread of lymph node metastases in patients with oral cancer. Int J Oral Maxillofac Surg 28: 279–284 10416895

[bib36] Silverman Jr S (2001) Demographics and occurrence of oral and pharyngeal cancers. The outcomes, the trends, the challenge. J Am Dent Assoc 132(Suppl): 7S–11S 11803655 10.14219/jada.archive.2001.0382

[bib37] Snow GB, Annyas AA, van Slooten EA, Bartelink H, Hart AA (1982) Prognostic factors of neck node metastasis. Clin Otolaryngol Allied Sci 7: 185–192 7105450 10.1111/j.1365-2273.1982.tb01581.x

[bib38] Sobin LH, Gospodarowicz MK, Wittekind C (eds) (2009) TNM Classification of Malignant Tumors, 7th edn, Wiley-Blackwell: New York

[bib39] Sunpaweravong P, Sunpaweravong S, Puttawibul P, Mitarnun W, Zeng C, Baron AE, Franklin W, Said S, Varella-Garcia M (2005) Epidermal growth factor receptor and cyclin D1 are independently amplified and overexpressed in esophageal squamous cell carcinoma. J Cancer Res Clin Oncol 131: 111–119 15672286 10.1007/s00432-004-0610-7 PMC12161209

[bib40] Takahashi K, Uzawa N, Myo K, Okada N, Amagasa T (2009) Simultaneous assessment of cyclin D1 and epidermal growth factor receptor gene copy number for prognostic factor in oral squamous cell carcinomas. Oral Sci Int 6: 8–20

[bib41] Uzawa N, Sonoda I, Myo K, Takahashi K, Miyamoto R, Amagasa T (2007) Fluorescence *in situ* hybridization for detecting genomic alterations of cyclin D1 and p16 in oral squamous cell carcinomas. Cancer 110: 2230–2239 17893905 10.1002/cncr.23030

[bib42] Wenzel S, Sagowski C, Kehrl W, Metternich FU (2004) The prognostic impact of metastatic pattern of lymph nodes in patients with oral and oropharyngeal squamous cell carcinomas. Eur Arch Otorhinolaryngol 261: 270–275 14504863 10.1007/s00405-003-0678-8

[bib43] Woolgar JA, Rogers SN, Lowe D, Brown JS, Vaughan ED (2003) Cervical lymph node metastasis in oral cancer: the importance of even microscopic extracapsular spread. Oral Oncol 39: 130–137 12509965 10.1016/s1368-8375(02)00030-1

[bib44] World Health Organization (1998) International Histological Classification of Tumors, 2nd edn, Springer: Berlin

[bib45] Xia W, Lau YK, Zhang HZ, Xiao FY, Johnston DA, Liu AR, Li L, Katz RL, Hung MC (1999) Combination of EGFR, HER-2/neu, and HER-3 is a stronger predictor for the outcome of oral squamous cell carcinoma than any individual family members. Clin Cancer Res 5: 4164–4174 10632356

[bib46] Yamamoto E, Kohama G, Sunakawa H, Iwai M, Hiratsuka H (1983) Mode of invasion, bleomycin sensitivity, and clinical course in squamous cell carcinoma of the oral cavity. Cancer 51: 2175–2180 6189571 10.1002/1097-0142(19830615)51:12<2175::aid-cncr2820511205>3.0.co;2-m

[bib47] Zhou X, Temam S, Oh M, Pungpravat N, Huang BL, Mao L, Wong DT (2006) Global expression-based classification of lymph node metastasis and extracapsular spread of oral tongue squamous cell carcinoma. Neoplasia 8: 925–932 17132224 10.1593/neo.06430 PMC1716013

